# Level-Specific Differences in Systemic Expression of Pro- and Anti-Inflammatory Cytokines and Chemokines after Spinal Cord Injury

**DOI:** 10.3390/ijms19082167

**Published:** 2018-07-25

**Authors:** James Hong, Alex Chang, Mohammad-Masoud Zavvarian, Jian Wang, Yang Liu, Michael G. Fehlings

**Affiliations:** 1 Division of Genetics and Development, Toronto Western Research Institute, University Health Network, Toronto, ON M5T 2S8, Canada; james.hong@live.com (J.H.); le.chang@mail.utoronto.ca (A.C.); Mohammad.zavvarian@mail.utoronto.ca (M.-M.Z.); jianw@uhnres.utoronto.ca (J.W.); Yang.Liu@uhnresearch.ca (Y.L.); 2 Institute of Medical Science, University of Toronto, Toronto, ON M5S 1A8, Canada; 3 Spinal Program, University Health Network, Toronto Western Hospital, Toronto, ON M5T 2S8, Canada

**Keywords:** spinal cord injury, inflammation, plasma

## Abstract

While over half of all spinal cord injuries (SCIs) occur in the cervical region, the majority of preclinical studies have focused on models of thoracic injury. However, these two levels are anatomically distinct—with the cervical region possessing a greater vascular supply, grey-white matter ratio and sympathetic outflow relative to the thoracic region. As such, there exists a significant knowledge gap in the secondary pathology at these levels following SCI. In this study, we characterized the systemic plasma markers of inflammation over time (1, 3, 7, 14, 56 days post-SCI) after moderate-severe, clip-compression cervical and thoracic SCI in a rat model. Using high-throughput ELISA panels, we observed a clear level-specific difference in plasma levels of VEGF, leptin, IP10, IL18, GCSF, and fractalkine. Overall, cervical SCI had reduced expression of both pro- and anti-inflammatory proteins relative to thoracic SCI, likely due to sympathetic dysregulation associated with higher level SCIs. However, contrary to the literature, we did not observe level-dependent splenic atrophy with our incomplete SCI model. This is the first study to compare the systemic plasma-level changes following cervical and thoracic SCI using level-matched and time-matched controls. The results of this study provide the first evidence in support of level-targeted intervention and also challenge the phenomenon of high SCI-induced splenic atrophy in incomplete SCI models.

## 1. Introduction

Traumatic spinal cord injury (SCI)—despite breakthroughs in pre-operative, surgical and post-operative care—continues to be a life-threatening injury, both acutely and chronically [1]. After primary mechanical injury, a dual-edged cascade of inflammatory and vascular events—collectively referred to as the secondary injury phase—ensues [2,3]. While it is difficult to determine the causative mechanism of secondary injury, several mechanisms including vascular disruption [4], glutamate excitoxicity [5,6], lipid peroxidation [7,8,9], blood-spinal-cord-barrier disruption [10,11,12] and ionic imbalance [13,14] have been the focus of therapeutic targeting. The ultimate consequence of these events is apoptosis, neuronal and axonal death, and de/dys-myelination manifesting as grey and white matter loss at the injury epicenter [1].

Preclinical SCI studies thus far, driven by post-operative care requirements and ease-of-use, have most commonly employed thoracic SCI (tSCI) models despite the increased prevalence and incidence of cervical SCI (cSCI) [15]. A central rationale for specifically investigating cSCI models is the appreciation that critical anatomical differences exist between the cervical and thoracic spinal cord resulting in different pathophysiological responses to injury and treatment [16]. For instance, the cervical spine is composed of smaller vertebrae with increased mobility, has increased central and peripheral vascular supply and flow, a higher gray-white matter ratio, and contains the neural circuitry crucial for respiration, forelimb motion, and sympathetic outflow to the heart. Pathophysiologically, the cervical gray matter vasculature has less pericyte coverage than the thoracic cord, resulting in a blood spinal cord barrier (BSCB) predisposed to increased permeability [10]. Further, in high-thoracic transection models of SCI, removal of spinal sympathetic preganglionic neurons from supraspinal control results in autonomic dysreflexia [17]. This in turn has been shown to instigate immunosuppressive effects—known as SCI-induced immune depression syndrome (SCI-IDS)—that stem directly from early splenocyte death and splenic atrophy due to acute and repeated chronic exposure to glucocorticoids and intrasplenic norepinephrine [18].

As cSCI has a direct neurological impact on cardiovascular function and peripheral immunity [19,20,21], we aimed to characterize the temporal profile (3–56 days) of vascular and inflammatory markers after cSCI and tSCI and elucidate any level-specific changes in their expression. Further, as robust spleen-mass changes were observed in the aforementioned transection studies on SCI-IDS, we also evaluated time and sham-normalized spleen-body weight ratios in our model.

## 2. Results

Of the 35 proteins surveyed in this study, 19 passed our initial filtering criteria, while 16 proteins that contained interpolated, extrapolated or out-of-range values were removed. All comparisons below are presented in order from thoracic to cervical.

### 2.1. Level-Specific Differences in Plasma Protein Levels after Cervical and Thoracic Laminectomy

**Figure 1:**
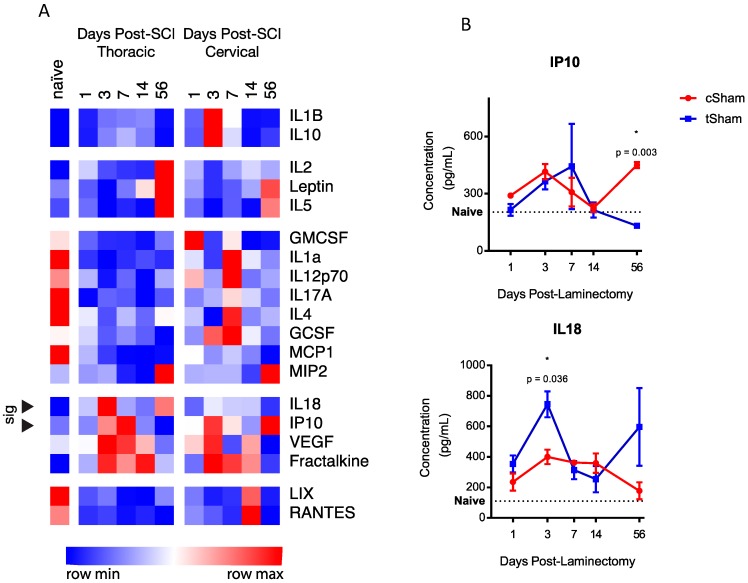
(**A**) Heat map and hierarchical cluster analyses reveal six clusters of temporal expression amongst cSham and tSham groups. Of the proteins analyzed, two proteins (marked with arrows) within cluster 4 reached statistical significance (IL18 and IP10). Naive data are shown as a baseline reference. Data are shown with relative color coding, with blue associated with the row minimum and red with the row maximum; all data are based on raw concentration in pg/mL; (**B**) Temporal expression of the two significant level-distinct proteins. Error bars represent SEM.

To investigate whether there were baseline differences in the expression of any of these proteins after cervical and thoracic laminectomy, heat-mapping and statistical analyses of protein concentrations were carried out with naïve plasma shown as a reference (excluded from cluster analyses). Heat-mapping (Figure 1A) demonstrated several clusters of expression. While several proteins had trending differences (RANTES, *p* = 0.06 at 14 days; LIX, *p* = 0.052 at 14 days; and IL10, *p* = 0.068), two proteins within cluster 5 showed significant differences in the expression of IP10 (56 days, 131.5 ± 11.2 vs. 450.3 ± 15.8 pg/mL, *p* = 0.003) and IL18 (3 days, 745.1 ± 84.8 vs. 400.2 ± 47.5 pg/mL, *p* = 0.036). The time-series expression of these three proteins is shown in Figure 1B.

### 2.2. Level-Specific Differences in Plasma Protein Levels after Cervical and Thoracic SCI

**Figure 2:**
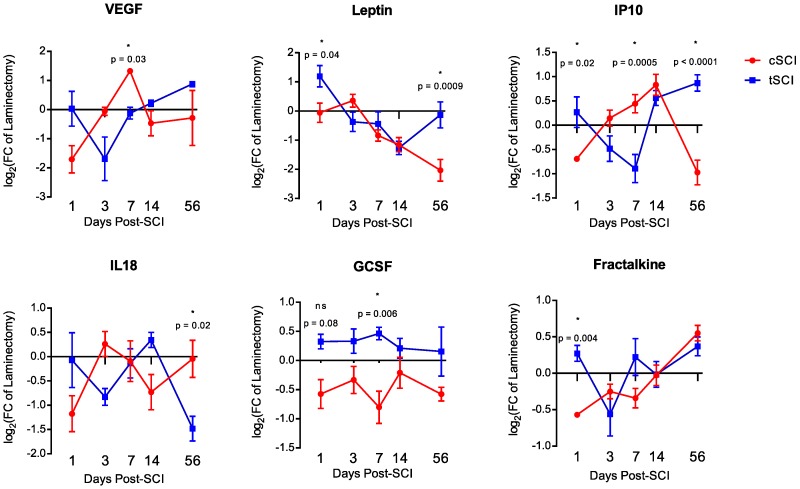
Temporal expression profile of the six significant differentially-expressed proteins: VEGF, leptin, IP10, IL18, GCSF and fractalkine. Error bars represent SEM.

Of the 19 proteins analyzed (Figure 2), six showed significant differences at one or more time-points between time-matched, level-matched, laminectomy-normalized cSCI and tSCI groups (expressed as fold-change to laminectomy). The six proteins that showed level-specific differences were VEGF (day 7, −0.1 ± 0.2 vs. 1.325 ± 0.1, *p* = 0.03), leptin (day 1, 1.2 ± 0.4 vs. −0.05 ± 0.3, *p* = 0.04; day 56, −0.1 ± 0.4 vs. −2.0 ± 0.4, *p* = 0.0009), IP10 (day 1, 0.3 ± 0.3 vs. −0.7 ± 0.04, *p* = 0.02; day 7, −0.891 ± 0.3 vs. 0.4 ± 0.2, *p* = 0.0005; day 56, 0.9 ± 0.2 vs. −1.0 ± 0.3, *p* < 0.0001), IL18 (day 56, −1.5 ± 0.3 vs. −0.05 ± 0.4, *p* = 0.02), GCSF (day 7, 1.010 ± 0.187 vs. 3.943 ± 1.663, *p* = 0.006), and fractalkine (day 1, 0.3 ± 0.1 vs. −0.6 ± 0.2, *p* = 0.004). Both of the proteins (IP10 and IL18) that showed level-specific baseline differences were significant after SCI. However, while differences in the 56-day baseline of IP10 contributed to a significant result, the 3-day baseline difference in IL18 did not.

### 2.3. Temporal Expression Patterns of Plasma Proteins after SCI

**Figure 3:**
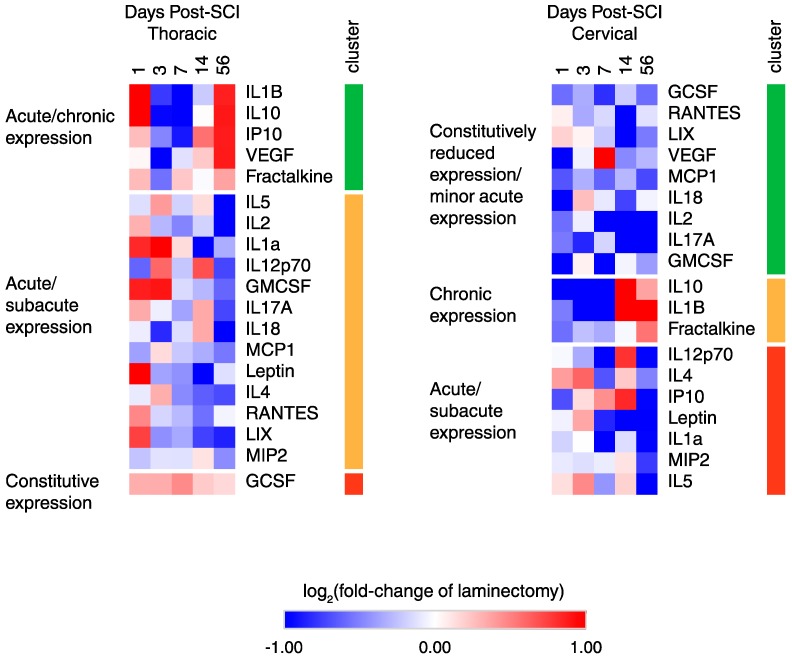
Heatmap and *k*-means clustering reveals three major clusters of temporal expression in cSCI and tSCI. Expression is displayed as log2 (fold-change of laminectomy) with blue indicating downregulation and red indicating upregulation.

To dissect the various temporal expression patterns after cSCI and tSCI, heatmap and *k-*means cluster analysis were performed (Figure 3). In tSCI, three main clusters were found with cluster 1 showing acute/chronic upregulation with subacute downregulation; cluster 2 showing an acute/subacute upregulation with chronic downregulation; and cluster 3 consisting of a single member showing constitutive upregulation. Similarly, in cSCI, three major clusters were defined with cluster 1 showing constitutive downregulation with some acute upregulation; cluster 2 showing proteins with acute/subacute downregulation with chronic upregulation; and cluster 3 showing proteins that had acute/subacute upregulation followed by chronic downregulation.

### 2.4. Functional Classification of Serum Protein after SCI

**Figure 4:**
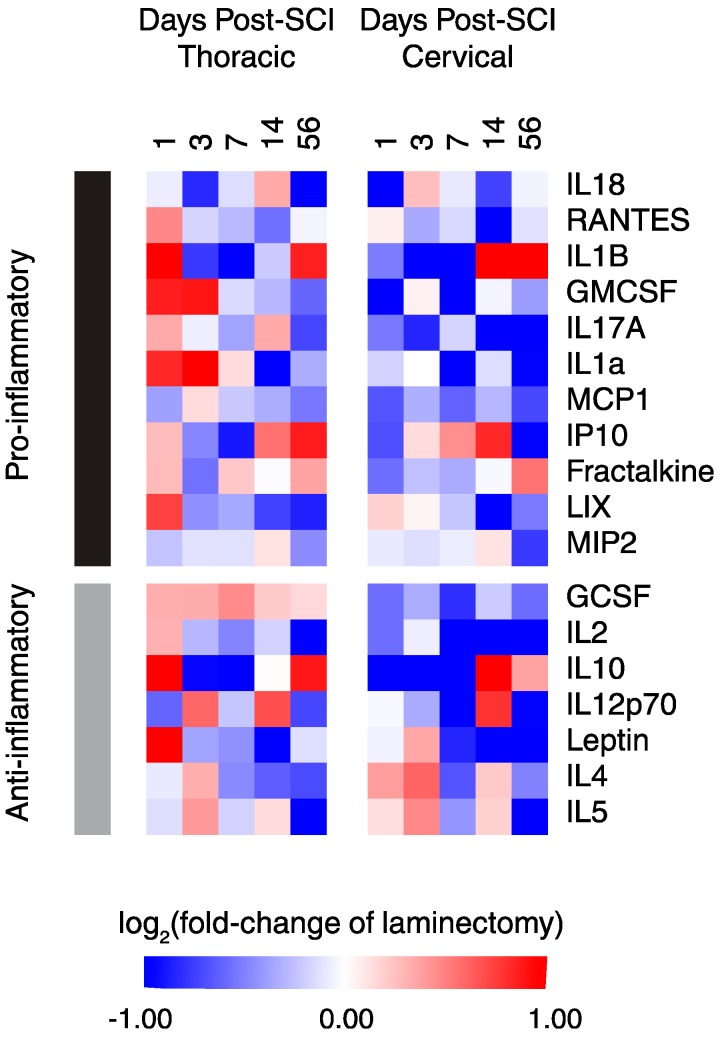
Heat map of functionally-segregated proteins after cSCI and tSCI. Expression is displayed as log2 (fold-change of laminectomy) with blue indicating downregulation and red indicating upregulation.

Using several reviews and meta-analyses articles [22,23,24,25,26,27,28,29], pro-inflammatory and anti-inflammatory functions were assigned to each of the 19 proteins to survey the overall inflammatory status after cSCI and tSCI (Figure 4). It is evident that overall, tSCI has increased expression of both pro- and anti-inflammatory proteins over time compared to cSCI. While most of these proteins are strikingly upregulated in the acute phase of thoracic relative to cervical SCI, a few of these differences equilibrated chronically (e.g., IL1b, fractalkine, IL10).

### 2.5. Spleen Weight

**Figure 5:**
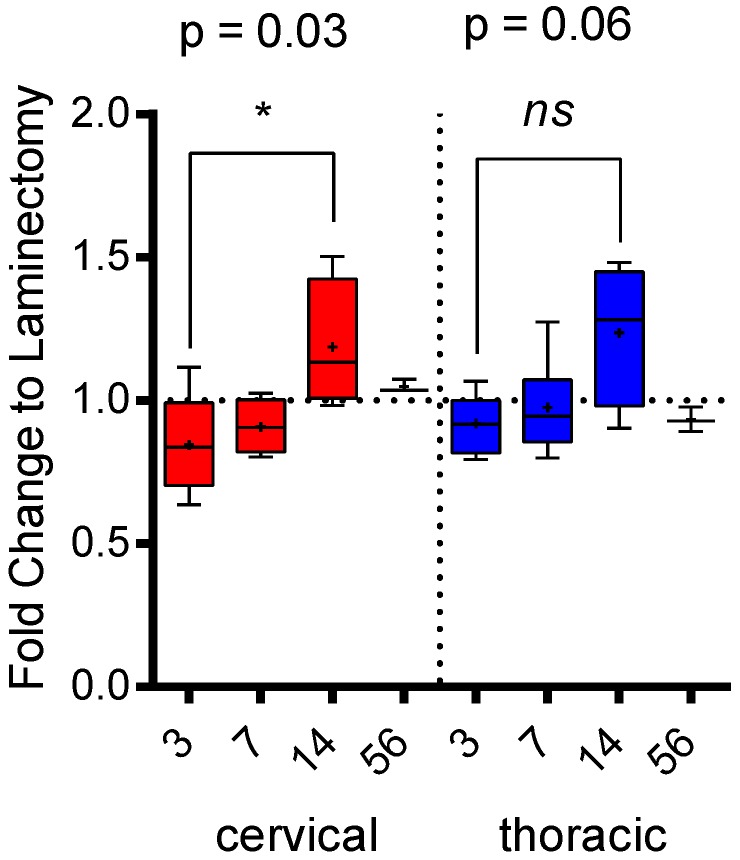
Mass: spleen ratios expressed as fold-change of time-matched laminectomized shams. Error bars represent SEM, and means are indicated by +. A significant change in spleen weight was observed in cSCI between day 3 and day 14.

To identify whether splenic atrophy was observed in our model of incomplete SCI, mass-normalized spleen weights were measured and expressed as a fold-change of time-matched laminectomized shams (Figure 5). While a decrease in weight between injured and time-matched laminectomized shams was seen in both cSCI and tSCI, this did not reach statistical significance. However, a significant increase in spleen weight was observed between 3 and 14 days in cSCI (0.862 ± 0.078 vs. 1.188 ± 0.113, *p* = 0.03), but only trended for tSCI (*p* = 0.06).

## 3. Discussion

In summary, this is the first study to characterize the temporal plasma expression profile of multiple cytokines, chemokines and growth factors after SCI. It is also the first study to use clinically-relevant models of cSCI and tSCI to determine level-specific differences in the expression of these inflammation-related molecules. This study establishes three main points: (1) time and level-matched laminectomy controls are essential for accurate data interpretation after SCI; (2) there exist both acute and chronic differences in plasma protein expression between cSCI and tSCI; and (3) splenic atrophy is not a robust phenomenon after incomplete cSCI, and as there continues to be evidence of peripheral immune depression after cSCI, it is also not a conclusive diagnostic tool for assessing the state of SCI-IDS.

Inflammation after SCI is considered one of the major drivers of secondary injury and tissue loss, and is often considered a dual-edged sword [30,31]. Our current results examined a small percentage of the cytokine/chemokines/growth factors involved, however, due to the spectrum of cells that secrete these factors, we cannot accurately pinpoint the cellular mediators of the temporal and level-specific changes that we have observed. With regards to the cause of level-differences between laminectomized shams, it is likely they are due to the degree of invasiveness and its associated fibrosis above the site of laminectomy and its differential impact on the cord over time between the two levels.

Studies on five of the six level-distinct proteins have already been conducted in the rodent tSCI model, with IL18 being the only exception with no SCI-associated studies. VEGF is well-known as a potent angiogenic factor that promotes the growth and development of endothelial cells. Our lab was one of the first to study the role of VEGF as a therapeutic agent after acute tSCI [32,33]. In these studies, transcriptionally-enhancing VEGF expression resulted in increased axon preservation, reduced necrosis, and an increase in blood vessels that ultimately translated to increased functional recovery as measured by Catwalk gait analyses. Another study using a contusion tSCI model [34], found that acute intraspinal infusion of VEGF into the lesion epicentre induced autophagy and reduced inflammation in the spinal cord, ultimately resulting in functional recovery as measured by the BBB motor scale. In this study, they showed that VEGF administration reduced the expression of IL1b, IL10 and TNFa in in vitro cultures of LPS-treated neuro-glia co-cultures. In our study, VEGF was upregulated at day 7 relative to tSCI and this change did indeed coincide with striking systemic reductions in IL1b and IL10. Further, an upregulation of these proteins was observed at 14- and 56-days post-cSCI when VEGF expression returned to baseline. Perhaps one of the major underlying factors of this level-dependent change after cSCI is the impact of cervical injury on the systemic vasculature as autonomic dysreflexia contributes directly to frequent vascular stress. Overall, there is significant evidence to suggest that VEGF therapy would be effective in both acute cSCI and tSCI, with the potential to also reduce chronic inflammation in both models.

As a hormone produced mainly by adipose cells, leptin is crucial for energetic balance in the central nervous system. Previous studies into leptin have shown that it is often upregulated both locally and systemically after tSCI [35,36,37]. While these acute studies were severely limited by the lack of time-matched controls, we observed a striking upregulation of plasma leptin in tSCI, but not cSCI at 1- and 56-days post-SCI. As leptin is regulated by the sympathetic nervous system, a study has shown that patients with high level SCIs have dysfunctional leptin expression [38]—thus supporting our data. A study [39] that acutely administered purified leptin in a rodent model of tSCI showed increased expression of neuroprotective genes, reduced inflammation and improved BBB, Catwalk and von Frey metrics suggesting that acute and chronic leptin deficiency may be a potent therapeutic target in SCI.

An upregulation in systemic and local IP10 has been demonstrated in both human and rodent SCI [40,41,42], and while no time-matched controls were used, this upregulation persisted as long as 14 days post-SCI in the murine tSCI model. IP10, is a chemokine secreted by a wide array of immune cells, endothelial cells and fibroblasts in response to IFNg [43]. In our study, IP10 expression was inversely expressed between the two levels, with cSCI experiencing a peak of expression during the subacute phase (days 3–14), and tSCI in the acute and chronic phases (day 1 and 56). Studies that neutralized the expression of IP10 showed markedly reduced inflammation, apoptosis, tissue loss and showed modestly improved BBB and BMS outcomes [42,44,45].

While GCSF has had no reported systemic or local expression in the SCI literature, here we find that the expression of GCSF is opposite in cSCI and tSCI. That is, while GCSF is upregulated in tSCI, it is downregulated in cSCI with time-to-time changes also in contrary motion with the exception of day 56. As purified GCSF administration alone and in combination with adipose- and bone-marrow-derived stem and neural stem cells has been shown to be highly beneficial in rodent and human tSCI—including increased tissue preservation, reduced apoptosis and scarring, and improved BBB, BMS and A [46,47,48,49,50,51,52,53,54,55,56]—such a paradigm may prove to be even more effective in the all stages of cSCI where a deficiency in GCSF is seen.

Receptor knockouts of the fractalkine receptor CX3CR1 have resulted in reduced iNOS^+^/Ly6C^low^/MHCII^+^/CD11c^−^ macrophages and activated microglia that have reduced expression of IL6 and iNOS. In these studies, the authors observed modest improvements in the BMS score [57,58,59]. In our study, the systemic fractalkine ligand is significantly upregulated in tSCI at 1-day post-SCI relative to cSCI, with both tSCI and cSCI experiencing late peaks 56-days post-SCI. Fractalkine is present in both a cell-bound and soluble form, and while both forms are potent chemo-attractants for migrating monocytes, the soluble form is also known to attract T cells. As such, while fractalkine receptor antagonism may be an ideal therapeutic target for acute tSCI, it may also be a valuable target for chronic tSCI and cSCI.

Two potential mechanisms by which cSCI induces an overall decrease in circulating protein are (1) SCI-IDS [20,21] and (2) increased cellular localization (and as such cytokine/chemokines) to the site of injury [26,28,41]. SCI-IDS is a phenomenon characterized by rapid splenic atrophy due to repeated bouts of autonomic dysreflexia in higher-level injuries. Interestingly, level-dependent splenic atrophy was not observed between our two incomplete models of cSCI and tSCI (Figure 5). The latter cytokine/chemokine “sink” concept is well-supported by the literature, as recent characterizations of cytokine/chemokine profiles in the spinal cord of SCI rodents and individuals show a striking acute and chronically-persistent expression of many pro- and anti-inflammatory cytokines. This, in conjunction with the increased BSCB permeability after cSCI, may well result in the formation of an inflammatory *milieu* that can be a potent trigger for secondary injury—especially chronic inflammation. All in all, we have shown striking evidence of level-specific differences in the systemic plasma expression of various cytokines and chemokines. In light of these results, preclinical researchers should adapt time-matched laminectomized controls and consider the impact of anatomical level on the therapeutic target of interest.

## 4. Materials and Methods

All animal experiments were approved by the Animal Care Committee of the University Health Network (Project ID Code: #2212, Date of Approval: 17 May 2017) in compliance with the Canadian Council on Animal Care.

### 4.1. Clip-Compression SCI and Spleen Weight

Female adult Wistar rats (12-weeks old, 250–300 g, *n* = 5/group for injured, *n* = 3/group for laminectomy and naïve) were used (Charles River Laboratories, Wilmington, MA, USA, http://www.criver.com). Prior to surgery, 0.05 mg/kg of buprenorphine and 5 mL of saline were administered subcutaneously. 1–2% of isoflurane in a 1:1 mixture of O_2_ and N_2_O was used for anesthesia, and a laminectomy was performed at C6-7 and T6-7, respectively. Following this, a moderate-severe injury was induced for 1-min at the cervical or thoracic level as described previously [15,60]. Until the endpoint (1, 3, 7, 14, 56 days post-SCI), the animals were given subcutaneous buprenorphine (0.05 mg/kg, bid), oral amoxicillin trihydrate/clavulanate potassium (Apotex Pharmaceuticals, Toronto, ON, Canada) and subcutaneous saline injections (0.9%, 5 mL sid). Animals were housed individually in cages at 27 °C, and their bladders were manually expressed thrice daily until recovery. Prior to sacrifice and perfusion, animal mass and spleens were collected from anesthetized rats and their weight recorded and normalized to their body mass.

### 4.2. Neurobehavioural Assessments

**Figure 6:**
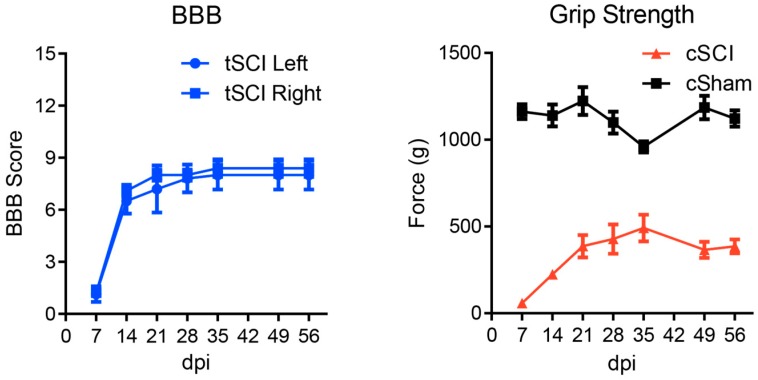
Hindlimb (BBB) and forelimb (Grip strength) assessments following tSCI and cSCI, respectively. No statistical outliers were detected using Grubb’s test (α > 0.05) indicating good homogeneity of data. Error bars represent SEM.

Starting at 7 days post-SCI, weekly forelimb and hindlimb function were assessed with the grip strength meter (SDI Grip Strength System DFM-10, San Diego Instruments, San Diego, CA, USA, http://www.sandiegoinstruments.com) and the BBB Locomotor Rating Scale [61] for cSCI and tSCI, respectively (Figure 6).

### 4.3. Blood Collection and High-Throughput ELISA

Blood was collected via a cardiac puncture prior to perfusion using a BD-Vacutainer^®^ Safety-Lok™ (Franklin Lakes, New Jersey, US) blood collection set containing EDTA. The blood samples were kept on ice and immediately centrifuged at 3000 rpm (Eppendorf 5810R) for 10 min at 4 °C. The plasma (supernatant) was then carefully aspirated and transferred to a Protein Lo-Bind tube (Eppendorf, Hamburg, Germany). 100 µL of the sample was then sent to Eve Technologies (Calgary, AB, Canada, https://www.evetechnologies.com) for high-throughput ELISA profiling using their rat Discovery Assays™ for cytokine/chemokines (RD27) and vascular injury markers (P1, P2). All proteins that contained interpolated/extrapolated/out-of-range values were removed from the study. The concentration of these proteins was calculated using a standard curve and expressed in pg/mL.

### 4.4. Clustering and Statistical Analysis

Data are presented as mean±SEM and comparisons are presented in order from cervical to thoracic. Heatmap, *k*-means and hierarchical row clustering (1−Cosine Similarity) was performed using the Morpheus software package from the Broad Institute (Cambridge, MA, USA, https://software.broadinstitute.org/morpheus/). Assessment of normality was performed for each group using the Shapiro-Wilk test of the *Rfit* package. All protein level comparisons between cSCI and tSCI were performed in GraphPad using either the one-way ANOVA function with post-hoc Sidak’s for multiple corrections (parametric, p-adjusted threshold = 0.05) or Krustal-Wallis test with post-hoc Dunn’s for multiple corrections (non-parametric, p-adjusted threshold = 0.05). Spleen-body mass ratio comparisons were done in GraphPad using a one-way ANOVA with post-hoc Sidak for multiple corrections (p-adjusted threshold = 0.05).

## Abbreviations

BBB	Basso, Beattie and Bresnahan
BMS	Basso Mouse Scale
BSCB	Blood-spinal-cord-barrier
PBS	Phosphate buffer solution
SCI	Spinal cord injury
GMCSF	Granulocyte-macrophage colony-stimulating factor
IL1a	Interleukin 1 alpha
IL1b	Interleukin 1 beta
IL2	Interleukin-2
IL17A	Interleukin-17A
IL18	Interleukin-18
MCP1	Monocyte chemoattractant protein-1
IP10	Interferon gamma-induced protein 10
LIX	Lipopolysaccharide-induced CXC chemokine
MIP2	macrophage inflammatory protein 2
RANTES	regulated on activation, normal T cell expressed and secreted
GCSF	Granulocyte-colony stimulating factor
IL4	Interleukin-4
IL10	Interleukin-10
IL12	Interleukin-12
IL5	Interleukin-5
IFNg	Interferon gamma
VEGF	Vascular endothelial growth factor
